# Transmission tree of the highly pathogenic avian influenza (H5N1) epidemic in Israel, 2015

**DOI:** 10.1186/s13567-016-0393-2

**Published:** 2016-11-04

**Authors:** Timothée Vergne, Guillaume Fournié, Michal Perry Markovich, Rolf J. F. Ypma, Ram Katz, Irena Shkoda, Avishai Lublin, Shimon Perk, Dirk U. Pfeiffer

**Affiliations:** 1 Veterinary Epidemiology, Economics and Public Health Group, Department of Production and Population Health, Royal Veterinary College, Hatfield, UK; 2 Veterinary Services, Ministry of Agriculture and Rural Development, Bet Dagan, Israel; 3 Brain Mapping Unit, University of Cambridge, Cambridge, UK; 4 Kimron Veterinary Institute, Bet Dagan, Israel

## Abstract

The transmission tree of the Israeli 2015 epidemic of highly pathogenic avian influenza (H5N1) was modelled by combining the spatio-temporal distribution of the outbreaks and the genetic distance between virus isolates. The most likely successions of transmission events were determined and transmission parameters were estimated. It was found that the median infectious pressure exerted at 1 km was 1.59 times (95% CI 1.04, 6.01) and 3.54 times (95% CI 1.09, 131.75) higher than that exerted at 2 and 5 km, respectively, and that three farms were responsible for all seven transmission events.

**Electronic supplementary material:**

The online version of this article (doi:10.1186/s13567-016-0393-2) contains supplementary material, which is available to authorized users.

## Introduction, methods and results

In mid-January 2015, the Israel’s national reference laboratory for avian influenza (Kimron Institute), confirmed the presence of highly pathogenic avian influenza (H5N1) virus in an extensive turkey farm. In an attempt to control the spread of the virus, human and poultry movements were restricted and culling, cleaning and disinfection were implemented in the infected farm and its vicinity. Within the next 4 weeks, the virus was isolated in seven other farms, mainly turkey farms, all located within 25 km from the first case. The objectives of this study were to estimate relevant transmission parameters and to reconstruct the most likely sequence of transmission events by combining the spatio-temporal distribution of the outbreaks and the genetic distance between the virus isolates.

The data used in this study relate to the eight cases of highly pathogenic avian influenza (H5N1) that were reported in Israel in January and February 2015. For all infected farms, the location, the date when increased mortality was reported, the date when samples were taken for laboratory confirmation and the date when cleaning and disinfection ended were recorded. Actual dates of infection were unknown and were therefore treated as model parameters to be estimated [1]. In each infected farm, a single virus strain was isolated and its full hemagglutinin gene was sequenced [2]. Assuming that the isolated strains were representative of the pool of viruses in the farms where they had been sampled, the genetic distance between virus isolates was determined.

The modelling approach used in this study combines epidemiologic and genetic data to infer possible transmission trees. It has already been used to model the spread of several animal pathogens, including highly pathogenic avian influenza virus [1, 3] and foot-and-mouth disease virus [4]. This approach assumes that all cases were reported and that there was only one virus introduction in the study area: except for the index case that had been infected by an unknown source, all successive cases were infected by one of the seven other infected farms through an unknown route.

To reconstruct the transmission tree, it was hypothesised that the likelihood that farm A infected farm B increased if A was still infectious when B became infected, if A and B were geographically close to each other, if the genetic sequence taken from A was similar to that from B and if there was no other farm that could have infected B.

Making the additional assumption of independence, the approximate likelihood L(AB) that farm A infected farm B is equal to the product of a temporal likelihood (L_t_(AB)), a spatial likelihood (L_s_(AB)) and a genetic likelihood (L_g_(AB)) as follows:

$$L\left( {AB} \right) = L_{t} \left( {AB} \right)*L_{s} \left( {AB} \right)*L_{g} \left( {AB} \right).$$

For the temporal likelihood, it was assumed that a farm became infectious one day after it became infected [5] and remained as such until the day when cleaning and disinfection ended. The temporal likelihood was therefore given by:

$$L_{t} \left( {AB} \right) = \left\{ {\begin{array}{*{20}l} {0 } \hfill & { \quad if \quad t_{inf} \left( B \right) \le t_{inf} \left( A \right)} \hfill \\ 1 \hfill & {\quad if \quad t_{inf} \left( A \right) < t_{inf} \left( B \right) \le t_{clean} \left( A \right)} \hfill \\ 0 \hfill & {\quad if \quad t_{inf} \left( B \right) > t_{clean} \left( A \right)} \hfill \\ \end{array} } \right.$$

with t_inf_(X) being the day when farm X became infected and t_clean_(X) being the day when cleaning and disinfection procedures ended in farm X at which point the farm was assumed not to be infectious anymore. As avian influenza viruses usually spread fast, a temporal resolution below one day could have been useful but the data were not recorded at that scale preventing the use of more temporally-precise models.

For the spatial likelihood, as often used in spatially explicit transmission models for avian influenza [3, 6], it was assumed that the likelihood that farm A infected farm B decreased with the distance between A and B. Two different functional forms were considered for the spatial likelihood, reflecting different assumptions as to how rapidly the likelihood decays with distance. These were:

$$L_{s1} \left( {AB} \right) \propto \frac{1}{{1 + d_{AB}^{\alpha } }}$$

and

$$L_{s2} \left( {AB} \right) \propto \frac{1}{{1 + \left( {\frac{{d_{AB} }}{\beta }} \right)^{\alpha } }}$$

with d_AB_ being the distance in kilometres between A and B and α and β being the parameters controlling the shape of the kernel.

Finally, for the genetic likelihood, it was assumed that, for each infection, each of the *N* nucleotides of the sequenced gene (here, *N* = 1643) could mutate with probability π. The genetic likelihood was therefore given by an ordered binomial distribution:

$$L_{g} \left( {AB} \right) \propto \pi^{{m_{AB} }} *(1 - \pi )^{{N - m_{AB} }}$$

with m_AB_ being the number of mutations between the strain isolated in farm A and that isolated in farm B. According to this ordered binomial probability distribution, the genetic likelihood of a transmission event is reduced by a factor (1 − π)/π for every additional mutation. Note that because the dataset comprised only eight cases, it was decided to use a one-parameter model for the genetic likelihood, in contrast to Ypma et al. [3] who used separate parameters for transitions and transversions. Also, because the data did not include any deletion, the parameter for deletion was not included in the genetic likelihood.

Because the dates of infection were unknown, they were treated as additional parameters to be estimated. Also, because information about non-infected farms was not available, the general likelihood function was normalised by dividing it by the sum of the product of the spatial and temporal likelihoods over all infectious farms. For any set of model parameters, the likelihood of a transmission tree can then be calculated by multiplying together the normalised likelihoods of all transmission events of that tree, one for each infected farm excluding the index case. As these events are independent, for each set of parameters we can compute the summed likelihood over the set *T* of all possible transmission trees as the product of the sums of the columns of the 7 × 8 matrix of likelihoods:

$$\mathop \sum \limits_{T} L(T|\pi ,\alpha ,t_{inf} ) = \mathop \prod \limits_{{B \in F^{*} }} \mathop \sum \limits_{A \in F} L(AB|\pi ,\alpha ,t_{inf} )$$

where *F* is the set of infected farms and *F* ^*^ is *F* minus the index case. Using a Bayesian approach, the posterior over all parameters was sampled using a Monte-Carlo Markov Chain algorithm. In light of the results presented in [3], the prior distributions that were used for the parameters of the spatial likelihood (α and β) were gamma distributions of mean 2.5 and variance 5. The prior of the parameter of the genetic likelihood (π) was given a uniform distribution between (0, 0.3) as negative values are not allowed and values larger than 0.3 were considered as highly irrelevant. Infection dates were given uniform priors between 4 and 8 days before reporting dates. Two simulation chains of 200 000 iterations were run, with the first 20 000 iterations discarded to allow for burn-in of the chain. The chains were then thinned, taking every thirtieth sample to reduce autocorrelation amongst the samples. Convergence of the chains was assessed by checking the trace plots for all monitored parameters. Comparison of the fit of the models using the different spatial likelihoods was done using the deviance information criterion (DIC) [7]. The best model was considered to be the most parsimonious model whose DIC was less than two points greater than that of the model associated with the smallest DIC.

The probability p(AB) for a potential transmission event from A to B was taken as the expected value of the transmission event (encoded as 1 or 0). Due to our assumption that all farms (except the index farm) were infected by another farm in the dataset, we calculated this expected value, for a given set of parameters θ, by dividing the likelihood of the transmission event L(AB|θ) by the sum of the likelihoods of all possible infection events for B. p(AB) was then calculated by taking the average of this value for all sampled parameters. These probabilities of the transmission events were then used to reconstruct the transmission tree of the most likely transmission events. To establish the posterior distribution of the effective reproduction number *R*_*i*_ for a given farm *i*, we calculated *R*_*i*_ for each set of parameters as the expected number of farms infected by it:

$$R_{i} = \mathop \sum \limits_{j \ne i} p\left( {i, j} \right).$$

**Figure 1:**
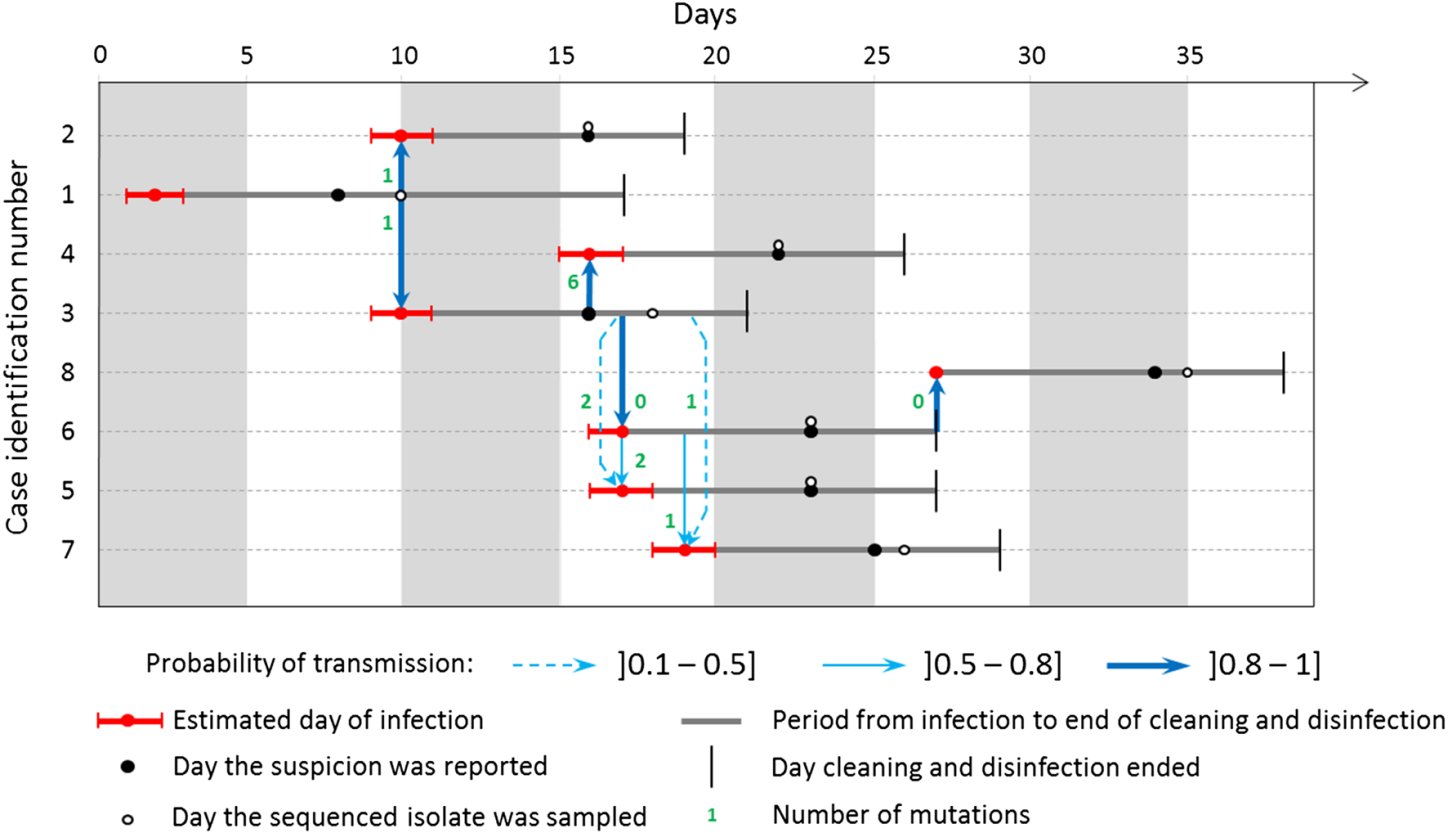
**Estimated trees of the most likely transmission events.** The case identification numbers (y-axis) represent the farms in chronological order of reporting (they were rearranged for the sake of figure clarity). Arrows represent transmission events whose probability was at least 0.1. Arrows are annotated with the number of mutations. Day 0 corresponds to the 6^th^ January 2015.

The best-fit model was the model using the one-parameter spatial likelihood L_s1_ (DIC = 191.2 versus 191.5). Estimated parameter values are presented in Table 1. The shape parameter of the spatial kernel, estimated at 1.12 (95% credible interval 0.11, 3.46), defines the relative likelihood of a transmission event between an infectious and a susceptible farm as a function of the distance between them: the median infectious pressure exerted at 1 km is expected to be 1.59 times (95% CI 1.04, 6.01) and 3.54 times (95% CI 1.09, 131.75) higher than that exerted at 2 and 5 km, respectively. The probability for a nucleotide to have mutated during a transmission event was estimated at 1.06e−3 (95% CI 0.55e−3, 1.82e−3), which is comparable with estimates provided in [3] for a different avian influenza subtype. For all transmission events but two, the infecting farm could be identified with probability greater than 0.9; this was true for all farms at probability 0.5 (Figure 1). The effective reproduction numbers were highly variable from negligible (<1e−2) for farm 2, 4, 5 and 7 to 2.00, (95% CI 2.00, 2.00), 2.89 (95% CI 1.18, 3.48) and 2.11 (95% CI 1.52, 3.82) for farms 1, 3 and 6, respectively. The transmission tree suggests that only three farms (farms 1, 3 and 6) were likely to be responsible for all seven transmission events. Note that the very small 95% credible interval for the effective reproduction number of farm 1 is due to the fact that the model predicted that farms 2 and 3 had almost a 100% chance to have been infected by farm 1 and that farm 1 had almost a 0% chance to have infected any other farm (because of the temporality of the cases and the genetic distance between isolates).

**Table 1:** **Summary of the posterior distributions of the parameters**

Parameter	Interpretation	Median (95% credible interval)
α	Shape parameter of the spatial kernel	1.12 (0.11, 3.46)
π	Probability of mutation	1.06e−3 (0.55e−3, 1.82e−3)
Re-farm 1	Effective reproduction number of farm 1	2.00 (2.00, 2.00)
Re-farm 2	Effective reproduction number of farm 2	Negligible (<1e−02)
Re-farm 3	Effective reproduction number of farm 3	2.92 (1.27, 3.48)
Re-farm 4	Effective reproduction number of farm 4	Negligible (<1e−02)
Re-farm 5	Effective reproduction number of farm 5	Negligible (<1e−02)
Re-farm 6	Effective reproduction number of farm 6	2.08 (1.52, 3.73)
Re-farm 7	Effective reproduction number of farm 7	Negligible (<1e−02)

## Discussion

At the time each farm (except the index case) was likely to become infected (i.e. between 4 and 8 days before reporting) there was at least one farm that was still infectious (already infected but not yet cleaned and disinfected) within a radius of 20 km. Therefore, the spatio-temporal distribution of the eight outbreaks does not show evidence that some outbreaks remained undetected or that there was more than one virus introduction. However, whilst six of the seven strains isolated amongst the secondary cases had two or less than two nucleotides of difference relative to at least one previously isolated strain, the strain isolated in farm 4 differed from all other previously isolated strains by at least six nucleotides. Possible reasons for this include (1) a sudden burst in mutations on farm 4, (2) the transmission of a very different subvariant from the farm that infected farm 4, (3) the presence of undetected infected farms that infected farm 4 or (4) a secondary introduction to farm 4. Further phylogenetic analyses would be required to assess the likelihood of a separate introduction [8], although these will be challenging to apply to the current dataset due to the small number of farms infected.

The strains sequenced on farms 4 and 7 displayed the same point mutation (position 132, see Additional file 1). Given that this mutation was not found in strains isolated from any other farms, it is unlikely to have occurred independently on both farms. This pattern may reflect infection from a common source: strains isolated on farms 4 and 7 might have both originated from a variant that appeared—but was not isolated—on farm 3 (Figure 1). Alternatively, this may also suggest a transmission event, not captured in the modelled transmission tree, between these two farms. Such a transmission event might have been direct between these two farms or mediated by unreported cases elsewhere. It is worth noting that the number of mutations between strains isolated in different farms had a strong influence on the estimated likelihood of the transmission events. Indeed, each additional mutation decreased the likelihood of the transmission event by a factor equal to the odds of the mutation rate, estimated here at 944 (95% CI 549, 1827). Consequently, to ensure meaningful inference, it is crucial to appreciate the genetic diversity of a strain within a farm by sequencing several strains from the same infected farm [5], and to integrate this information into the transmission tree modelling. Until then, such analyses should be interpreted cautiously [4].

**Figure 2:**
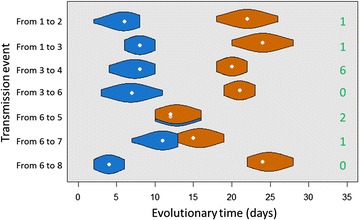
**Violin plots of the minimum (blue) and maximum (orange) evolutionary times for each most likely transmission event.** The minimum evolutionary time is the sum of the time between infection of the receiver farm and the sampling of the infector farm and the time between infection of the receiver farm and the sampling of the receiver farm. Assuming a transmission viral population bottleneck of size 1, the maximum evolutionary time is the sum of the time between infection of the infector farm and the sampling of the infector farm and the time between infection of the infector farm and the sampling of the receiver farm. The numbers in green represent the genetic distance associated to the corresponding transmission event.

To more accurately model the genetic distance between isolated strains, we could have accounted for the evolutionary time separating the samples taken from an infected farm and its infector. On these short time scales, we could reasonably assume that the expected number of mutations increases linearly with the evolutionary time separating sequences. Such a model extension would yield maximal discriminatory power when times between sequences show high variance. However, as is evident from Figures 1 and 2, the evolutionary times between sequenced isolates are fairly constant throughout the outbreak. Thus, adding evolutionary time to our genetic likelihood is unlikely to have strong impact on inference, as was previously found for a similar (but larger) outbreak of avian influenza [9]. Given the small number of transmission events available here, we deemed this extension to not merit the associated increase in model complexity, and followed [3] in omitting evolutionary time.

Whilst most of the likely transmission events identified using the transmission tree modelling were consistent with outbreak investigations, the former approach cannot incorporate as many sources of information as the latter to make informed decisions and is therefore more limited when it comes to unexpected transmission events, particularly with small datasets. A continuation of this work could be to incorporate the prior knowledge on transmission events generated from the outbreak investigations into the Bayesian parameter estimation procedures to estimate integrated measures of transmission probabilities.

Transmission tree modelling provided a consistent statistical framework to investigate the 2015 Israeli HPAI (H5N1) epidemic. By combining spatial, temporal and genetic data, it was possible to estimate transmission parameters and reconstruct the sequence of the most likely transmission events under a set of assumptions. We suggest that such a statistical approach should be used in real time to gain additional insights into the evolution of an epidemic. We further note that sequencing several strains isolated in each infected farm will allow better capturing genetic diversity and aid in calibrating and validating such models.

## Additional file

**Additional file 1.** **Sequence alignment.** Comparative alignment of the 8 isolates is presented.
