# Supplementary material for: Transmission tree of the highly pathogenic avian influenza (H5N1) epidemic in Israel, 2015
Source: Vet Res. 2016 Nov 4;47:109. doi: 10.1186/s13567-016-0393-2 (PMC5096331; doi:10.1186/s13567-016-0393-2)
Supplement: Supplementary file 1 — Additional file 1. Sequence alignment. Comparative alignment of the 8 isolates is presented. [file 13567_2016_393_MOESM1_ESM.pdf]

```
           10        20        30        40        50        60        70        80        90       100
   ....|....|....|....|....|....|....|....|....|....|....|....|....|....|....|....|....|....|....|....|
1  ATCAGATTTGCATTGGTTACCATGCAAACAACTCGACAGAGCAGGTTGACACAATAATGGAAAAGAACGTCACTGTTACACACGCCCAAGACATACTGGA
2  ....................................................................................................
3  ....................................................................................................
4  .........................................................................................A..........
5  ....................................................................................................
6  ....................................................................................................
7  ....................................................................................................
8  ....................................................................................................

          110       120       130       140       150       160       170       180       190       200
   ....|....|....|....|....|....|....|....|....|....|....|....|....|....|....|....|....|....|....|....|
1  AAAGACACACAACGGGAAACTCTGCAATCTAGATGGAGTGAAGCCTCTCATTTTAAGAGATTGTAGTGTAGCTGGATGGCTCCTCGGGAACCCAATGTGC
2  ....................................................................................................
3  ....................................................................................................
4  ...............................A.................................A....A.............................
5  ....................................................................................................
6  ....................................................................................................
7  ...............................A....................................................................
8  ....................................................................................................

          210       220       230       240       250       260       270       280       290       300
   ....|....|....|....|....|....|....|....|....|....|....|....|....|....|....|....|....|....|....|....|
1  GATGAATTCCTCAATGTGCCGGAATGGTCTTACATAGTGGAGAAAATCAATCCAGCCAATGACCTCTGTTATCCAGGGAATTTCAACGACTATGAAGAAC
2  ....................................................................................................
3  ....................................................................................................
4  ................................................................................................A...
5  ....................................................................................................
6  ....................................................................................................
7  ....................................................................................................
8  ....................................................................................................

          310       320       330       340       350       360       370       380       390       400
   ....|....|....|....|....|....|....|....|....|....|....|....|....|....|....|....|....|....|....|....|
1  TGAAACACCTATTGAGCAGAATAAACCATTTTGAGAAAATTCAGATCATTCCCAAAGATTCTTGGTCAGATCATGAAGCCTCAGGAGTGAGCTCAGCATG
2  ....................................................................................................
3  ........T...........................................................................................
4  ........T.........T........................T........................A...............................
5  ........T...........................................................................................
6  ........T...........................................................................................
7  ........T...........................................................................................
8  ........T...........................................................................................

          410       420       430       440       450       460       470       480       490       500
   ....|....|....|....|....|....|....|....|....|....|....|....|....|....|....|....|....|....|....|....|
1  CCCATACCAAGGAAGATCCTCCTTTTTTAGAAATGTTGTATGGCTTACCAAAAAGAACGATGCATACCCAACAATAAAGAAAAGTTACAATAATACTAAC
2  ....................................................................................................
3  ....................................................................................................
4  ....................................................................................................
5  ....................................................................................................
6  ................................G.......................................................T...........
7  ....................................................................................................
8  ....................................................................................................

          510       520       530       540       550       560       570       580       590       600
   ....|....|....|....|....|....|....|....|....|....|....|....|....|....|....|....|....|....|....|....|
1  CAAGAAGATCTTTTGGTACTATGGGGGATTCACCATCCAAATGATGCTGCAGAGCAGACAAGGCTTTATCAAAACCCAACTACCTATATCTCCGTTGGGA
2  ....................................................................................................
3  ....................................................................................................
4  ....................................................................................................
5  ....................................................................................................
6  ....................................................................................................
7  ....................................................................................................
8  ....................................................................................................

          610       620       630       640       650       660       670       680       690       700
   ....|....|....|....|....|....|....|....|....|....|....|....|....|....|....|....|....|....|....|....|
1  CATCAACACTAAACCAGAGATTGGTACCCAAAATAGCTACTAGATCTAAGGTAAACGGGCAAAGTGGAAGGATGGAGTTCTTTTGGACAATTTTAAAATC
2  ....................................................................................................
3  ....................................................................................................
4  ....................................................................................................
5  ....................................................................................................
6  ....................................................................................................
7  ....................................................................................................
8  ....................................................................................................
```

```
          710       720       730       740       750       760       770       780       790       800
     ....|....|....|....|....|....|....|....|....|....|....|....|....|....|....|....|....|....|....|....|
1    GAATGATGCAATAAACTTTGAGAGCAATGGAAACTTCATTGCTCCAGAAAATGCATACAAAATTGTCAAGAAAGGAGATTCAACAATTATGAAAAGTGAG
2    ....................................................................................................
3    ....................................................................................................
4    ....................................................................................................
5    ....................................................................................................
6    ....................................................................................................
7    ....................................................................................................
8    ....................................................................................................

          810       820       830       840       850       860       870       880       890       900
     ....|....|....|....|....|....|....|....|....|....|....|....|....|....|....|....|....|....|....|....|
1    TTGGAATATAGTAACTGCAACACCAAGTGTCAGACTCCAATAGGGGCGATAAACTCCAGTATGCCATTCCACAACATCCACCCTCTCACCATCGGGGAAT
2    ....................................................................................................
3    ....................................................................................................
4    ....................................................................................................
5    ....................................................................................................
6    ....................................................................................................
7    ....................................................................................................
8    ....................................................................................................

          910       920       930       940       950       960       970       980       990      1000
     ....|....|....|....|....|....|....|....|....|....|....|....|....|....|....|....|....|....|....|....|
1    GCCCCAAATATGTGAAATCAAACAGATTAGTTCTTGCTACTGGGCTCAGGAATAGCCCTCAAGGAGAGAAAAGAAGAAAAAAGAGAGGACTATTCGGAGC
2    ....................................................................................................
3    ....................................................................................................
4    ....................................................................................................
5    ....................................................................................................
6    ....................................................................................................
7    ....................................................................................................
8    ....................................................................................................

         1010      1020      1030      1040      1050      1060      1070      1080      1090      1100
     ....|....|....|....|....|....|....|....|....|....|....|....|....|....|....|....|....|....|....|....|
1    CATAGCAGGCTTTATAGAGGGAGGATGGCAGGGAATGGTAGATGGTTGGTATGGGTACCACCATAGCAACGAGCAGGGGAGTGGGTACGCTGCAGACAAA
2    ....................................................................................................
3    ....................................................................................................
4    ....................................................................................................
5    ....................................................................................................
6    ....................................................................................................
7    ....................................................................................................
8    ....................................................................................................

         1110      1120      1130      1140      1150      1160      1170      1180      1190      1200
     ....|....|....|....|....|....|....|....|....|....|....|....|....|....|....|....|....|....|....|....|
1    GAATCCACTCAAAGGGCTATAGATGGAGTCACCAATAAGGTCAATTCGATCATTGACAAAATGAACACTCAGTTTGAGGCTGTTGGAAGGGAATTTAATA
2    ...........................A........................................................................
3    ....................................................................................................
4    ....................................................................................................
5    ....................................................................................................
6    ....................................................................................................
7    ....................................................................................................
8    ....................................................................................................

         1210      1220      1230      1240      1250      1260      1270      1280      1290      1300
     ....|....|....|....|....|....|....|....|....|....|....|....|....|....|....|....|....|....|....|....|
1    ACTTAGAAAGGAGAATAGAAAATTTAAACAAGAAGATGGAAGACGGATTCCTAGATGTCTGGACTTATAATGCTGAACTTCTGGTTCTCATGGAAAATGA
2    ....................................................................................................
3    ....................................................................................................
4    ....................................................................................................
5    ....................................................................................................
6    ....................................................................................................
7    ....................................................................................................
8    ....................................................................................................

         1310      1320      1330      1340      1350      1360      1370      1380      1390      1400
     ....|....|....|....|....|....|....|....|....|....|....|....|....|....|....|....|....|....|....|....|
1    GAGAACTCTAGACTTTCATGACTCAAATGTCAAGAATCTTTATGACAAGGTCCGACTACAGCTTAGGGATAATGCAAAGGAGCTTGGTAACGGTTGTTTC
2    ....................................................................................................
3    ....................................................................................................
4    ....................................................................................................
5    ....................................................................................................
6    ....................................................................................................
7    ....................................................................................................
8    ....................................................................................................
```

```
           1410      1420      1430      1440      1450      1460      1470      1480      1490      1500
       ....|....|....|....|....|....|....|....|....|....|....|....|....|....|....|....|....|....|....|....|
1      GAGTTCTATCACAGATGTGATAATGAATGTATGGAAAGTGTAAGAAACGGAACGTATGACTACCCTCAATATTCAGAAGAAGCAAGATTAAAAAGAGAGG
2      ....................................................................................................
3      ....................................................................................................
4      ....................................................................................................
5      ....................................................................................................
6      ....................................................................................................
7      ....................................................................................................
8      ....................................................................................................

           1510      1520      1530      1540      1550      1560      1570      1580      1590      1600
       ....|....|....|....|....|....|....|....|....|....|....|....|....|....|....|....|....|....|....|....|
1      AAATAAGTGGAGTAAAATTGGAGTCAATAGGAACTTACCAAATACTGTCAATTTATTCAACAGTGGCGAGCTCCCTAGCACTGGCAATCATGGTGGCTGG
2      ....................................................................................................
3      ....................................................................................................
4      ....................................................................................................
5      ....................................................................................................
6      ....................................................................................................
7      ....................................................................................................
8      ....................................................................................................

           1610      1620      1630      1640
       ....|....|....|....|....|....|....|....|...
1      TCTATCTTTATGGATGTGCTCCAATGGATCGCTACAATGCAGA
2      ...........................................
3      ...........................................
4      ...........................................
5      ...........................................
6      ...........................................
7      ...........................................
8      .....................G...................AG
```
